# Poly (Vinyl Alcohol)/Agar Hydrogel Electrolytes Based Flexible All-in-One Supercapacitors with Conducting Polyaniline/Polypyrrole Electrodes

**DOI:** 10.3390/polym14214784

**Published:** 2022-11-07

**Authors:** Khadija Hasan, Shahid Bashir, Ramesh Subramaniam, Ramesh Kasi, Kashif Kamran, Javed Iqbal, Hamed Algarni, Abdullah G. Al-Sehemi, S. Wageh, M. Pershaanaa, Fathiah Kamarulazam

**Affiliations:** 1 Centre for Ionics Universiti Malaya, Department of Physics, Faculty of Science, Universiti Malaya, Kuala Lumpur 50603, Malaysia; 2 Higher Institution Centre of Excellence (HICoE), UM Power Energy Dedicated Advanced Centre (UMPEDAC), Level 4, Wisma R&D, Universiti Malaya, Jalan Pantai Baharu, Kuala Lumpur 59990, Malaysia; 3 Department of Physics, University of Agriculture, Faisalabad 38040, Pakistan; 4 Center of Nanotechnology, King Abdulaziz University, Jeddah 21589, Saudi Arabia; 5 Research Center for Advanced Materials Science (RCAMS), King Khalid University, P.O. Box 9004, Abha 61413, Saudi Arabia; 6 Department of Physics, Faculty of Science, King Khalid University, P.O. Box 9004, Abha 61413, Saudi Arabia; 7 Department of Chemistry, College of Science, King Khalid University, P.O. Box 9004, Abha 61413, Saudi Arabia; 8 Department of Physics, Faculty of Science, King Abdulaziz University, Jeddah 21589, Saudi Arabia; 9 K. A. CARE Energy Research and Innovation Center, King Abdulaziz University, Jeddah 21589, Saudi Arabia

**Keywords:** agar, all-in-one flexible hydrogel, flexible, conducting polymers, supercapacitors

## Abstract

The major components of supercapacitor are electrodes and electrolytes which are fabricated using various materials and methods. Hydrogel is one such material that is used in supercapacitors as electrodes and electrolytes or both. Hydrogels are usually described as a soft and porous network of polymer materials that can swell in water because of the hydrophilic nature of its polymer chains, compriseng a 3D structure. It is well known that supercapacitors possess high-power density but low energy density. For enhancing energy density of these electrochemical cells and a boost in its electrochemical performance and specific capacity, binder free conducting polymer hydrogel electrodes have gained immense attention, especially polyaniline (PANI) and polypyrrole (PPy). Therefore, in this work, chemically crosslinked PVA/Agar hydrogel electrolytes have been prepared and employed. Agar has been added in PVA since it is environmentally friendly, biodegradable, and cost-effective natural polymer. Subsequently, the binder free polyaniline/polypyrrole electrodes were grown on the PVA/Agar hydrogel electrolytes to fabricate all-in-one flexible hydrogels. The synthesized hydrogels were characterized using X-ray diffraction (XRD) analysis, Fourier transform infrared (FTIR) analysis, Field emission scanning electron microscope (FESEM) and mechanical studies. Then, the all-in-one flexible supercapacitors were fabricated using the hydrogels. The electrochemical studies such cyclic voltammetry (CV), galvanic charge discharge (GCD), and electrochemical impedance spectroscopy (EIS) studies. The fabricated all-in-one lamination free supercapacitors showed promising results and by comparing all four samples, PAP2 where 5 mL of PVA was used in combination with 3 mL of Agar and 5 mL of PANI and PPy each, exhibited the highest areal capacitance of 750.13 mF/cm^2^, energy density of 103.02 μWh/cm^2^, and 497.22 μW/cm^2^ power density. The cyclic stability study revealed the 149% capacity retention after 15,000 cycles.

## 1. Introduction

As fossil fuels are non-renewable, therefore, the energy obtained from them is very precious with a challenging sustainability. Hence, many countries are now looking forward to adopting models to conserve energy and find substitutes that would also reduce the energy emissions and its impact on the environment. Energy can be stored using energy storage devices like batteries and supercapacitors, while can be utilized upon requirement. Batteries, however, show few disadvantages; for example, they are sensitive to overcharging, which can shorten their life and are costly.

A supercapacitor is an energy storage device having the tendency to charge faster, but the same goes for discharging. They possess long lifetime and render safety, which have been extensively implemented in digital electronics, electrical vehicles, and different power systems [1,2]. It can also be used in wearable and portable electronics and gadgets. Usually, wearable electronic gadgets available today use either batteries to get power or use a capacitor with a configuration of soft packing because these two energy storage devices are observed to be promising, however, they need to be long lasting and mechanically strong [3]. Supercapacitors have different approaches to store energy. Thus, these devices are classified into three types depending upon the approach they use for storing energy. These are namely electric double layer capacitors (EDLCs), pseudocapacitors, and hybrid capacitors. 

In the configuration of supercapacitors based on EDLC phenomenon, different forms of carbon have been employed as electrodes, for instance, graphene, carbon nanotubes, and activated carbon. However, electrolytes used are generally aprotic solvents-based liquid, which means that their molecules are without hydrogen-oxygen bonds or hydrogen-nitrogen. For example, propylene carbonate, ethylene carbonate and/or dimethyl carbonate. 

Pseudocapacitors are also known as Faradaic capacitors and are commercially minimally used in comparison to the EDLCs. Their working principle is near to the working principle of batteries. In pseudocapacitors, conductive polymer, transition metal oxide, or some other pseudo material is used as electrode/active material [4]. The energy storing phenomena happen on the electrodes’ surface because faradaic reactions occur on it and the energy passes across the double layer. This is similar to the discharging and charging of a battery. Pseudocapacitors experience redox reactions and electron exchange during the charging and discharging processes. However, there lies a disadvantage that, during the charging and discharging, the electrodes undergo a stress, so they deteriorate quickly. Their charging efficiency and discharge rate are low, with cyclability and stability being lower than the EDLCs.

Electrolytes are a significant part of a supercapacitor configuration since they allow the transfer of charges between the two electrodes. An electrolyte could only satisfy a supercapacitor’s electrochemical performance when it has lower viscosity, a good ionic concentration, a large potential window, and great stability. However, the liquid electrolytes in many cases cause inconvenience; for example, there is a high ionic mobility in liquid electrolytes, which causes the charges to redistribute in diversely shaped pores. This leads to the rapid self-discharging of the supercapacitor, which is the main drawback. Since there is always a risk of the corrosive electrolyte leakage from the system, it is necessary to properly encapsulate the system to avoid leakage, which could automatically increase the cost. Moreover, this typical configuration of supercapacitor is also a hindrance in its additional applications since it causes the supercapacitor to become heavier in weight [5]. It is therefore, indeed, necessary to replace the liquid electrolytes with an alternative electrolyte. Solid polymer electrolytes have also been reported in different research works and are synthesized by directly dissolving lithium salts like LiCF_3_SO_3_, LiN(SO_2_CF_3_)_2_, LiPF_6_, LiBF_4_, and LiClO_4_ in ion coordinating polymers like polyethylene glycol (PEG) and polyethylene oxide (PEO). Solid-polymer electrolytes-based configurations guarantee that supercapacitors working on this configuration would be able to supply as well as store electrical energy and would also have enough room to accumulate a certain volume of transformation. However, solid polymer electrolytes have poor ionic conductivity, which ultimately reduce the electrochemical performance of the fabricated supercapacitor. 

In the recent past, gel polymer electrolytes have been largely utilized in energy storage devices [6]. Polyvinyl alcohol (PVA) and polyethylene oxide (PEO) are the most frequently employed polymers in supercapacitors [7,8]. However, supercapacitors using these gel electrolytes have displayed weak electrochemical performance; and therefore, facing challenges because the liquid equivalents at least exhibit better ionic conductivity and less resistance. Also, the usage of corrosives like H_2_SO_4_, H_3_PO_4_ and LiCl are involved in implementing the formerly mentioned polymers [9]. 

Recently, modification in the supercapacitor electrolytes has been made by employing bio-sourced polymers among which hydrocellulose, chitosan, sodium alginate, gelatin, and chitin have been the center of attention as gelling agents. One interesting polymer is Agar, which has been reportedly employed in some research as gel electrolyte. Agar is a naturally occurring biodegradable as well as a biocompatible polymer, which is obtained from sea weeds particularly from red algae and is a fine gelling agent [9]. The structure of Agar consists of two important constituents, namely agarose (polysaccharide) and agaropectin (a heterogenous mixture of minor particles). Some of the properties of Agar include that it requires a temperature higher than 90 °C to get dissolve in water and only at temperatures below 45 °C it can solidify. The hydrogels that are produced using Agar show high elasticity and have a distinctive pore structure/size. Agar undergoes gelation owing to hydrogen bonding and enables the self-assembly of ions.

Menzel et al. [9] synthesized symmetric carbon/carbon supercapacitors with Agar acting as a gel electrolyte. The results showed a positive effect on hydrogen sorption/desorption, along with the lessening of internally built pressure and self-discharging of the capacitor to a considerable extent. Also, they assumed that the lessening of self-discharge occurred because of the viscoelastic characteristics of the gel electrolyte and the increased conductivity was a result of chemical composition. Moon et al. [5] synthesized NaCl/Agar gel electrolyte for flexible supercapacitors by simply adding a pair of sodium and chloride ions into a crosslinked Agar gel matrix followed by heating and then casting procedure. This is an easy, cost effective and non-toxic method. Since NaCl and Agar both are edible, therefore, this electrolyte combination is quite environmentally benign. The specific capacitance obtained from the fabricated supercapacitor at a scan rate of 5 mVs^−1^ was 200 Fg^−1^.

Metal oxides are employed as electrode material in supercapacitors. However, since they show low capacitance and their utilization in the energy storage devices is limited. Conducting polymers have recently gained noticeable consideration as an electrode material and, to be specific, much of the attention is being gathered by the polyaniline (PANI) and polypyrrole (PPy). Usually, pseudocapacitors show unique benefits like high specific capacitance, permanent flexibility, and comparatively higher conductivity [10,11].

Conducting polymer, polyaniline and polypyrrole are much cheaper and can be merely synthesized by uncomplicated methods. Conducting polymer hydrogels are generally prepared in a two-step method. Initially, non-conducting hydrogel is synthesized and then dried. This dried hydrogel matrix is then submerged into a monomer solution where it can polymerize. Recently, the self-assembly method has been tested and utilized to prepare conducting polymer hydrogels which also elevates its use as a method to prepare supercapacitor electrode material. However, conducting polymer hydrogel still lacks hence their effective and simple synthesis is still necessarily required [12,13].

The in situ growth of conducting polymer over hydrogel electrolyte is known as all-in-one configured supercapacitor. The advantage of all-in-one configuration supercapacitors is that they reduce the contact resistance between the electrolyte and the electrode to a great extent and results in improved energy densities. This configuration also helps in simplifying the assembling process of the devices [14,15]. Another considerable advantage is that, under deformations, this configuration prevents the delamination of the device along with its displacement [16]. Yu and co-workers fabricated an all-in-one flexible supercapacitor with a hydrogel electrolyte synthesized using acrylic acid monomers in PVA-H_2_SO_4_ solution. The designed device restored 80% of its capacitance even after it was stored for 23 days at a temperature of −35 °C. This showed that the fabricated device also has the potential to be operated in cold regions. It showed high mechanical strength while being stretched under a pressure of 25 kPa [17,18]. Y. Guo et al. developed a healable supercapacitor with an all-in-one configuration using the in situ polymerization method to deposit the nanocomposite electrode material onto a self-healing hydrogel electrolyte which was made up of PVA [17]. The overall device performance at a current density of 0.044 mA cm^−2^ showed an enhanced capacitance of 15.8 mF cm^−2^. Guo et al., developed an all-in-one flexible supercapacitor where poly(pyrrole-co-aniline) electrodes were in situ grown on a PVA/PEG hydrogel electrolyte. The device exhibited a specific capacitance of 773 mF cm^−2^ at a current density of 0.2 mA cm^−2^ [19].

Herein, the objectives of the study were to synthesize PVA/Agar hydrogel electrolytes, followed by in situ growth of polyaniline/polypyrrole as electrode material on the hydrogel electrolyte. The synthesized hydrogel electrolyte and all-in-one hydrogels were physico-chemically characterized using FTIR, XRD, and field emission scanning electron microscope (FESEM). The mechanical strength was investigated through the tensile testing. A flexible all-in-one configuration supercapacitor was then fabricated by sandwiching the hydrogels in graphite sheets. Moreover, electrochemical performance of the all-in-one configured supercapacitor was investigated through cyclic voltammetry (CV), galvanic charge discharge (GCD), and electrochemical impedance spectroscopy studies. In addition, the electrochemical stability of the optimized supercapacitor cell was investigated. 

## 2. Methodology

### 2.1. Materials

Poly (vinyl alcohol) (PVA, 87–90% hydrolysed, Mol. Wt. 30,000–70,000), glutaraldehyde (GA, 25%), ammonium persulfate (APS ((NH_4_)_2_S_2_O_8_), Mol. Mass = 228.18 g/mol), ≥98.0%), and pyrrole monomer were purchased from Sigma Aldrich Co., USA. Agar was purchased from Lab M Limited, Lancashire, UK. Sulphuric acid (H_2_SO_4_, 98%) was obtained from EMSURE, UK. Aniline monomer was purchased from R&M Chemicals, Essex, UK. Graphite sheet was employed, and distilled water was used in all experiments.

### 2.2. Methods

**Figure 1:**
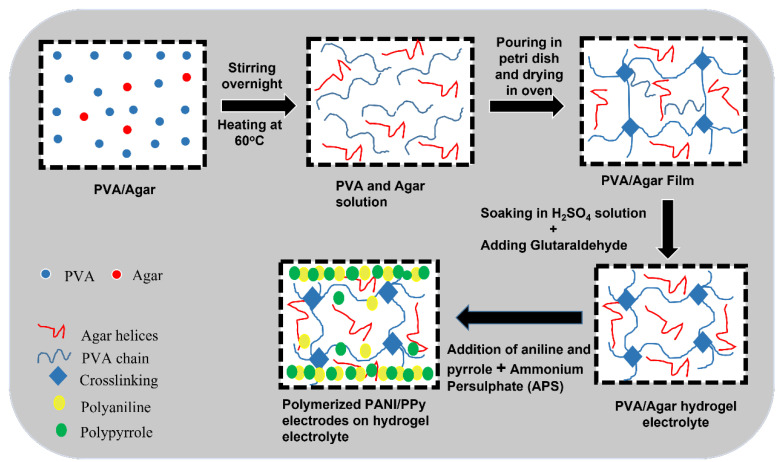
Illustration of the formation of electrolytes and electrodes.

To prepare the hydrogel electrolytes, 5 mL of 5 wt.% PVA solution was added in four beakers. Then, in these PVA solution containing beakers, 3 mL of 5 wt.% Agar solution was added in two beakers and 5 mL in other two beakers. All four beakers were kept on magnetic stirring at a temperature of 60 °C overnight so that PVA and Agar can be thoroughly incorporated into each other. The following day, all the four beakers were emptied in four different petri dishes which were then placed in a conventional oven for the solvent evaporation at 50 °C for 1 h. After the solvent evaporation, quite stable hydrogels were developed in which 2 mL of 1 M H_2_SO_4_ was added in each petri dish. Then, 1 mL of 4 wt.% Glutaraldehyde (GA) as a crosslinking agent was added in all the four petri dishes to form the hydrogels. The petri dishes were then covered with a parafilm. Polymers in their natural state do not show conductivity; therefore, the added sulphuric acid acts as a catalyst and a charge carrier. Glutaraldehyde is used because it favors intermolecular reaction with PVA and can crosslink the polymers. After few minutes, thin hydrogel films were formed which served as electrolytes. The synthesis steps are given in Figure 1.

#### In Situ Preparation of Binder Free PANI-PPy Electrodes

In situ polymerization of aniline and pyrrole was carried out in the presence of hydrogel electrolytes to form all-in-one hydrogels. For this purpose, 5 mL of aniline solution (1 M) and 5 mL of pyrrole solution (1 M) were added to the hydrogel electrolytes in two petri dishes out of four. Similarly, in the remaining two petri dishes, 2.5 mL of aniline solution (0.1 M) and 2.5 mL of pyrrole (0.1 M) solution were added. In addition to this, 2 mL of 1 wt.% ammonium persulfate (APS) was added in all four petri dishes as an initiator. All these petri dishes were then kept inside a refrigerator for the reaction to occur and for the better results. After the in situ polymerization of aniline and pyrrole that happened on both surfaces of the hydrogel electrolytes, the deposited layer served as PANI/PPy electrodes. This polymerization that occurred on the hydrogel surfaces was the actual in situ growth of the electrode material. The as-prepared all-in-one configured hydrogel-based supercapacitors were named as PAP1, PAP2, PAP3, and PAP4. The synthesis scheme is presented in Table 1.

### 2.3. Characterizations

The synthesized all-in-one configured hydrogels were cut into small pieces and were subjected to the freeze-drying process, after which XRD, FTIR, and morphology studies were carried out. Here the freeze-drying was executed at SupermodulY0230 freeze-dryer (ThermoFisher). The chemical structure of the synthesized all-in-one hydrogels was analyzed using FTIR-ATR (Nicolet^TM^ Summit Spectrometer (Thermo Scientific)) from 4000 to 500 cm^−1^ at a resolution of 1 cm^−1^ at ambient temperature. FTIR spectra were recorded in transmittance mode. The X-ray diffraction analysis of PVA film, PVA/Agar hydrogel electrolyte, PANI/PPy (5 mL each), PAP1, PAP2, PAP3, and PAP4 was carried out using PANalytical EMPYREAN diffractometer with Cu Kα radiation at 30 mA and 40 kV voltage. All the samples were rinsed with distilled water before the XRD analysis. Surface morphology of the hydrogels was observed by field emission scanning electron microscope (SU8220, Hitachi), keeping the accelerating voltage at 5 kV. Prior to the FESEM analysis, all the samples were first coated with platinum to enhance the conductivity of the samples to take high resolution images. Cross section of the hydrogels was obtained by cutting them along the radial direction. Uniaxial monotonic tensile testing was conducted at room temperature on Shimadzu AGS-X series equipped with 500 N load cell. Hydrogels were cut into rectangular shapes with the dimensions of length 4 cm and width 1.5 cm. Tensile tests were performed at a crosshead speed of 2 mm min^−1^ until failure.

### 2.4. Electrochemical Studies

**Figure 2:**
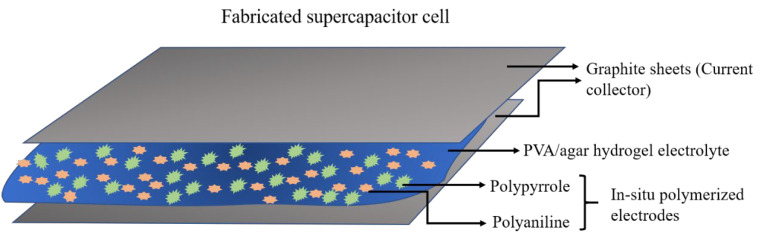
Graphical illustration of the fabricated all-in-one configuration supercapacitor cell.

The prepared all-in-one PANI/PPy-(PVA/Agar)-PANI/PPy hydrogels were then cut into rectangular shapes and the dimensions were 20 mm width × 20 mm length × 3 mm thickness. All-in-one supercapacitor cells were fabricated by sandwiching the all-in-one hydrogels in the graphite sheets. For this, graphite sheets which could act as current collectors were cut into rectangular pieces of the desired size. In this way, four complete binder free all-in-one-configuration supercapacitor cells were fabricated as shown in Figure 2.

To carry out the electrochemical studies, the fabricated supercapacitor cells were then connected with the Potentiostat (Gamry Interface-1000) one by one, where the graphite sheets served as current collector. The electrochemical performance of the device was carried out by using a two-electrode system. Cyclic voltammetry (CV) and galvanostatic charge discharge (GCD) analyses were carried out to investigate the capacitive behavior of the fabricated supercapacitors. CV was carried out at different scan rates of 5–100 mV/s at a potential window ranging from 0–1 V. Galvanostatic charge-discharge (GCD) of the supercapacitor cells was carried out at different current densities i.e., 0.5 mA/cm^2^ to 5 mA/cm^2^ at 1 V. This was followed by calculating the specific capacitance of the supercapacitor cells along with energy density and power density.

The specific capacitance, energy density, and power density were found by using the following Equation (1), Equation (2), and Equation (3), respectively:

(1) $$C_{sp}\left(\mathrm{GCD}\right)=\frac{2\times \;I\;\times \;t}{A\;\times \Delta V}$$

where I is the discharge current, t is the discharging time, A is the area of current collector and ΔV is the voltage change during the discharging time.

(2) $$E\;=\frac{1}{2}\times {\;C}_{\mathrm{sp}}\left(\mathrm{GCD}\right)\times {\left(\Delta V\right)}^{2}\times \frac{1000}{3600}$$

where $C_{\mathrm{sp}}$ is the calculated specific capacitance using GCD data and ΔV is the potential.

(3) $$P=\frac{E}{\Delta t}$$

where E is the obtained energy density and Δt is the discharging time.

An electrochemical impedance spectroscopy study (EIS) was examined with a frequency range of 0.01 Hz to 1 × 10^6^ Hz and a perturbation amplitude of 10 mV. The cyclic stability study of the optimized PAP2 was conducted at a current density of 3 mA/cm^2^ for 6000 consecutive charge discharge cycles.

## 3. Results and Discussion

### 3.1. Synthesis of Hydrogel Electrolytes and Conducting Polymer Electrodes

**Figure 3:**
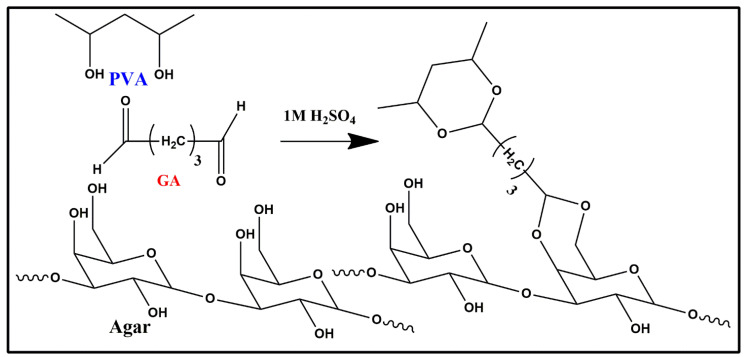
The mechanism occurred among PVA, Agar and glutaraldehyde.

The synthesis of flexible PVA/Agar hydrogel electrolyte formation took place initially with the magnetic stirring of PVA and Agar in the four beakers at a temperature of 60 °C overnight for a thorough incorporation of PVA and Agar into each other. After 24 h, well-incorporated solutions of PVA/Agar were obtained. However, these solutions are completely non-conductive since these polymers are non-conductive in nature. Therefore, sulphuric acid was added as a charge carrier in the PVA/Agar solutions to provide ionic conductivity to the resulting polymer hydrogel electrolytes as well as a catalyst to promote crosslinking to form hydrogel electrolytes. Following this, glutaraldehyde was added as a crosslinking agent to crosslink the polymer chains of PVA and Agar in the presence of 1 M H_2_SO_4,_ as shown in Figure 3. The mechanism which occurs behind the gelation is that poly (vinyl alcohol) and Agar both contain alcohol (hydroxyl (–OH) groups) in their structure while glutaraldehyde contains aldehyde (-CHO) group. Under the catalysis of sulphuric acid, the aldehyde groups of glutaraldehyde (GA) react with the (–OH) groups of poly (vinyl alcohol) (PVA) and Agar via chemical crosslinking to form an acetal or hemiacetal, which resulted in the gelation of PVA/Agar solution and three-dimensional hydrogels were formed which were obtained as electrolytes [20].

**Figure 4:**
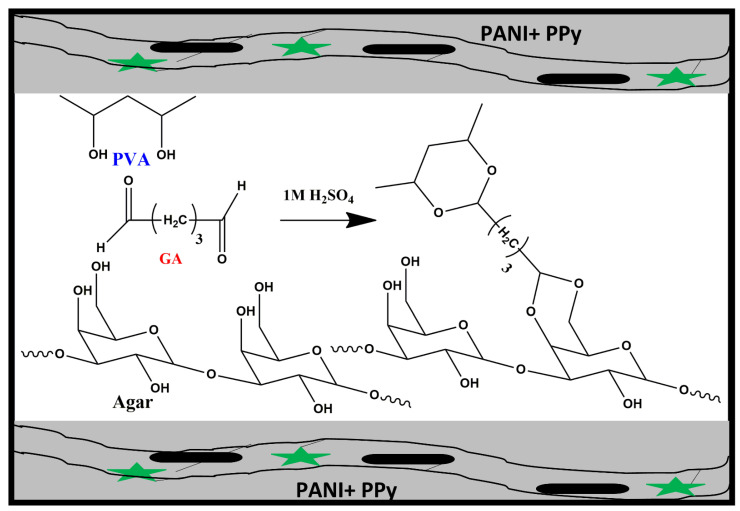
The in situ polymerization mechanism of electrode material (Top and bottom chains represent the PANI + PPy where green stars show PANI and bold lines reveal PPy).

For the in situ polymerization of aniline and pyrrole as electrode material, aniline solution and pyrrole solution which were prepared by adding the respective monomers in H_2_SO_4_ aqueous solution followed by an ultrasonic treatment, were added in the previously mentioned amount to the obtained hydrogel electrolytes, in the presence of ammonium persulphate (APS). These monomers initially infiltrated the PVA/Agar electrolyte layer after which they began to polymerize via chemical oxidation polymerization, to produce an embedded layer of electrodes which assists in the transport of electrons and ions along with reducing the interface resistance. Ultimately, polyaniline and polypyrrole were grown on both surfaces of the hydrogel electrolytes and served as PANI/PPy electrodes, as shown in Figure 4. In this way, an all-in-one configured PANI/PPy-(PVA/Agar)-PANI/PPy hydrogels were obtained which were flexible because of binder free non-laminated configuration.

### 3.2. Characterizations

#### 3.2.1. X-ray Diffraction

**Figure 5:**
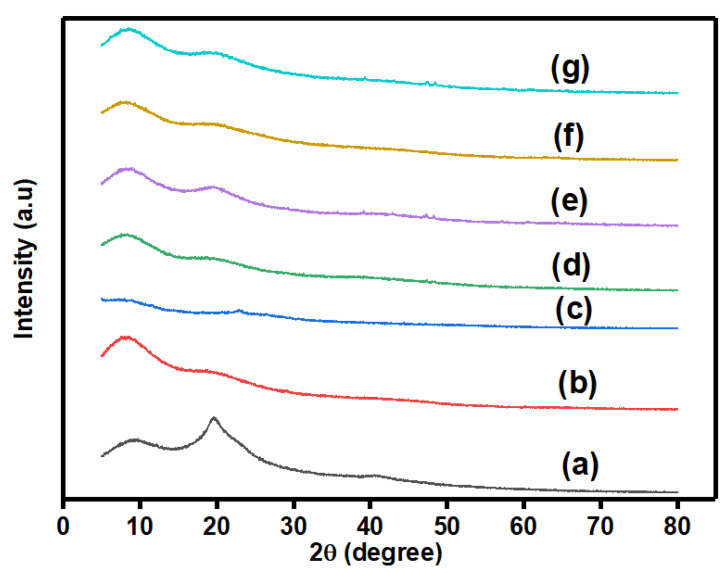
XRD diffractograms of (**a**) PVA, (**b**) (PVA/Agar)/H_2_SO_4_ hydrogel electrolyte, (**c**) PANI/PPy, (**d**) PAP1, (**e**) PAP2, (**f**) PAP3, and (**g**) PAP4.

To identify the crystalline/amorphous nature of PVA, (PVA/Agar)/H_2_SO_4_ hydrogel electrolyte, PANI/PPy, and all-in-one hydrogels, X-ray diffraction (XRD) analysis was carried out and results are presented in Figure 5a–g. The diffractogram of PVA has two broad peaks at 2θ = 9.6° and 19.7° representing the interaction among active sites and main chain of PVA, respectively. Such diffraction patterns indicate more trend towards amorphous nature of PVA. These peaks slightly shifted to 2θ = 8.5° and 19.6° upon adding Agar to PVA and hydrogel electrolyte formation in the presence of glutaraldehyde. The addition of Agar and hydrogel formation resulted in the decrement in peak intensity and increase in the amorphous characteristics. This significantly verifies the crosslinking and hydrogel formation. Moreover, PANI/PPy shows two less intense peaks at 2θ = 9° and 23°. In situ growth of aniline and pyrrole in the presence of hydrogel electrolytes resulted in all-in-one hydrogels such as PAP1, PAP2, PAP3, and PAP4. Herein, two broad, but very less intense peaks compared to (PVA/Agar)/H_2_SO_4_ hydrogel electrolyte can be observed, which are almost identical to (PVA/Agar)/H_2_SO_4_ hydrogel electrolyte. The reduction in intensity of the peaks reveals the successful in situ growth of PANI/PPy over hydrogel electrolyte and development of physical interactions between electrode and electrolyte. PANI/PPy and hydrogel electrolytes peaks are in the same range, therefore, there is no separate identification. However, it can be seen that peak positions and broadness in PAP1 and PAP3 are same and peak at 8.5° is slightly more intense compared to PAP2 and PAP4. The reason might be owing to higher content of Agar in the former than the latter all-in-one hydrogels. The large contents of Agar did not have complete miscibility with the PVA and showed the less amorphous trend. 

#### 3.2.2. Fourier Transform Infrared Analysis

**Figure 6:**
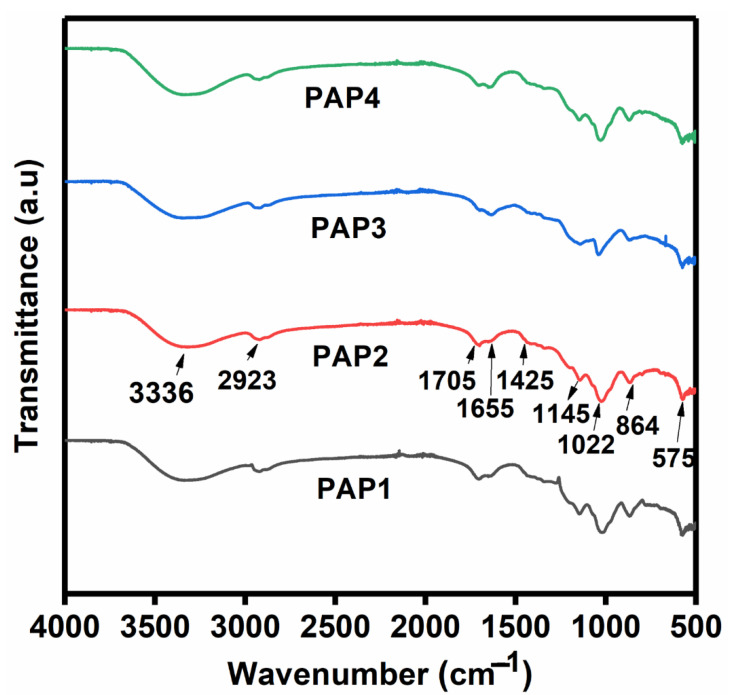
FTIR spectra of the synthesized all-in-one hydrogels.

Fourier transform infrared spectroscopy analysis was conducted to investigate the structural identification of functional groups present in the all-in-one hydrogels and to classify the chemical groups present in the lyophilized PANI/PPy-(PVA/Agar)-PANI/PPy hydrogels and FTIR spectra are shown in Figure 6. The peaks found around 3336 cm^−1^ correspond to the stretching vibrations of –OH indicating the presence of a large number of OH groups. The peak at 2923 cm^−1^ shows C–H bonds of PVA. The peaks at 1705 cm^−1^ and 1655 cm^−1^ are attributed to the C=C stretching vibrations of the quinoid ring and the benzenoid ring of PANI [19]. These peaks indicate the presence of PVA as well as PANI. Peak at 1425 cm^−1^ shows benzene in phenyl ring and C-N stretching vibration in pyrrole ring. Peak located around 1145 cm^−1^ was found because of the crosslinking occurred between PVA and Agar through glutaraldehyde (GA). It is designated to the ether (C–O) and acetal ring (C–O–C). This presents the evidence of GA reacting with PVA and Agar to form covalent bonds [20]. The peaks at 1022 cm^−1^ and 575 cm^−1^ show C-H in-plane bending on 1,2,4-subsituted benzene of PANI. The peak around 864 cm^−1^ shows C-H out-of-plane ring deformation vibration in pyrrole. 

#### 3.2.3. Surface Morphology

**Figure 7:**
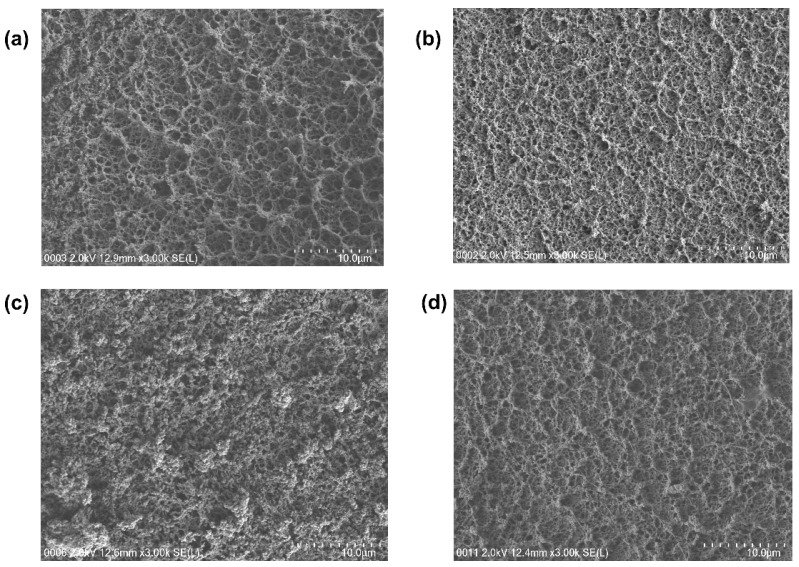
Surface morphology—FESEM micrographs (**a**): PAP1 (**b**) PAP2 (**c**) PAP3 (**d**) PAP4.

**Figure 8:**
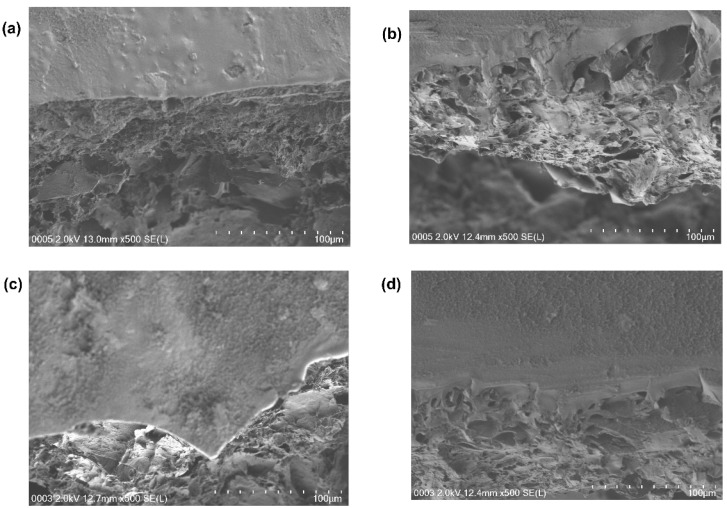
Cross section images of (**a**) PAP1 (**b**) PAP2 (**c**) PAP3 (**d**) PAP4.

The surface morphology and the cross-section of the freeze-dried hydrogels were characterized by field emission scanning electron microscopy (FESEM) and images are shown in Figure 7 and Figure 8, respectively. The samples were observed to possess a sponge like and a granular morphology with a hierarchal pore size and showed a porous 3D structure which are interconnected but also appear rough and uniformly covered with PANI/PPy. It is established that PANI/PPy have been evenly grown on both surfaces of PVA/Agar hydrogel electrolyte. Since PANI/PPy-(PVA/Agar)-PANI/PPy has a sandwich like configuration i.e., electrode-electrolyte-electrode, so to understand this configuration clearly, a cross-section of the lyophilized hydrogels was performed and studied. 

The FESEM cross-sectional images of the hydrogels show a continuous and clear electrode/electrolyte interface where a smooth electrode surface can be seen with a porous structure underneath which demonstrates flawless integration of PANI/PPy layer into the PVA/Agar hydrogel (Figure 8). Hence, this special configuration of flexible supercapacitors helps in facilitating the ion and electron transport along with eliminating the dislocations of electrode and electrolyte under large deformations [19]. 

#### 3.2.4. Tensile Testing

**Figure 9:**
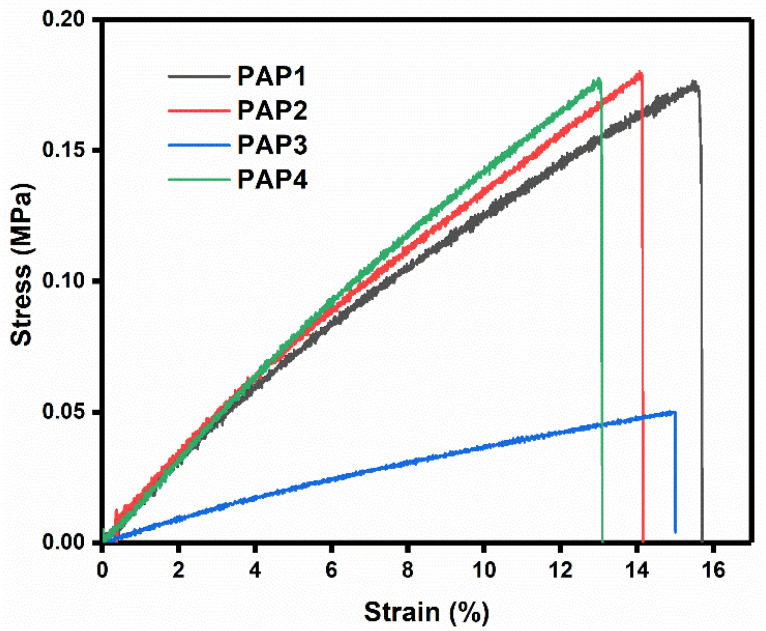
Tensile properties of the All-in-One hydrogels.

The as synthesized hydrogels were capable of withstanding various deformations, for instance, curving, folding and a cylindrical shape. It is important to note that GA crosslinking agent resulted in the rapid formation of PVA/Agar hydrogels in the presence of H_2_SO_4_. GA improved the mechanical strength through the formation of covalent crosslinking and physical interactions such as hydrogen bonding within the PVA/Agar network. Therefore, the tensile properties of the all-in-one flexible PANI/PPy-(PVA/Agar)-PANI/PPy hydrogels were investigated with a tensile testing machine. Tensile measurements were performed by uniaxially stretching the strips of hydrogel films and results are presented in the Figure 9. Hydrogels demonstrate the significant tensile modulus and tensile modulus are comparable in all the hydrogels. However, there is a slight influence on the modulus of the hydrogels, which was dependent on the composition of the hydrogel. The hydrogel containing more amount of Agar attained optimum at lower strain of 13.2% while the less Agar containing hydrogel achieved optimum at higher strain 15.8%. This optimum at lower strain% revealed the decrease in mechanical strength of the hydrogels, which is owing to the inherited weak mechanical properties of the natural polysaccharide Agar. 

### 3.3. Electrochemical Analysis

#### 3.3.1. Cyclic Voltammetry

PANI/PPy-(PVA/Agar)-PANI/PPy based all-in-one configuration hydrogel was sandwiched between two graphite sheets and this electrochemical cell was then connected with the Gamry Interface (1000). Cyclic voltammetry scans were carried out at a potential window ranging from 0 to 1 V at different scan rates of 5, 10, 20, 30, 40, 50, 60, 70, 80, 90 and 100 mV/s in order to characterize the electrochemical performance of the synthesized hydrogel electrolytes since it is a well-grounded analytical technique that is capable of providing information regarding the kinetics of charge carriers and their thermodynamics related to the provided electrolyte material to be used in supercapacitors. A two-electrode configuration has been used to carry out the measurements of the electrochemical performances. 

**Figure 10:**
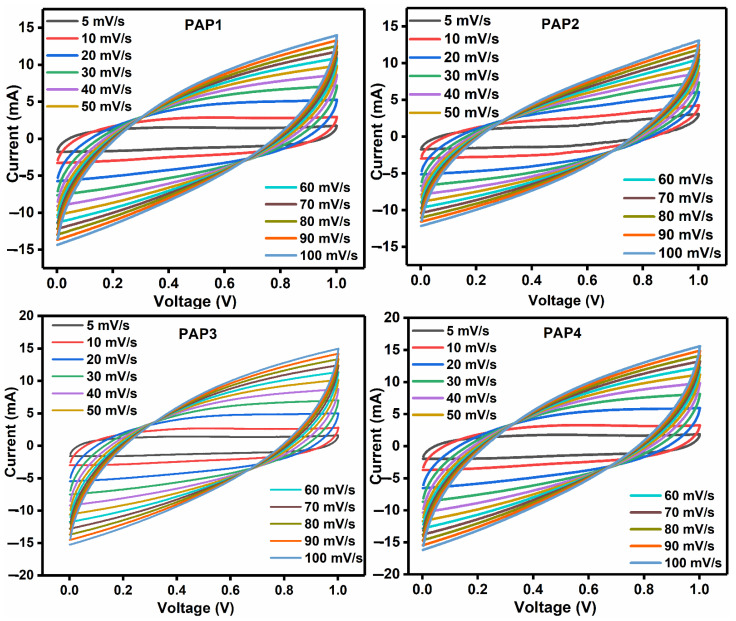
CV curves of all samples at different scan rates.

Figure 10 shows the CV curves of supercapacitors based on all-in-one configuration with PVA/Agar hydrogel electrolytes and in situ polymerized PANI/PPy electrodes in a potential range of 0–1 V. Here, the shape of the CV curves is indicating the proof of the associated electrochemical processes in the charging and discharging of the supercapacitor cells. The areal capacitance is depending on the contents of electrode and electrolyte used to prepare all-in-one hydrogels, and other factors like resistance to ion transport along with the speed at which ion diffusion occurs and scan rate of the CV analysis. The CV curves for all the cells demonstrate rectangular shape at small scan rate; however, the CV shapes became leaf-like upon increasing the scan rate. The areal capacitance decreased at higher scan rates. The leaf-like shapes and reduction in capacitance at higher scan rates are owing to the incomplete interaction between electrodes and electrolyte and the fast movement of ions at the interfacial boundary between electrode and electrolyte.

The cyclic voltammetry analysis explains the effect of PVA to Agar ratio and electrode materials contents (PANI/PPy) on the performance of all-in-one flexible supercapacitors. 

Other than the scan rate dependent CV analysis, the composition of PAP1, PAP2, PAP3, and PAP4 affected the capacitance of the supercapacitor cells. Among these cells, PAP2 comprising of 5 mL PVA and 3 mL Agar in hydrogel electrolyte while 5 mL aniline and pyrrole each as electrode material showed the most promising combination since it exhibited the highest area which indicates that the highest areal capacitance is shown by supercapacitor fabricated using PAP2. Therefore, the best results are achieved when with 5 mL of PVA and 3 mL of Agar combination with 5 mL of aniline and pyrrole solutions. 

Second highest specific capacitance is shown by PAP4 supercapacitor, which also contains 3 mL Agar, but electrode materials were polymerized using 2.5 mL of aniline and pyrrole solution, each unlike PAP2. So, by comparing these results, it shows that Agar when used in 3 mL amount has a promising effect on the electrochemical properties of the device because despite a different amount of aniline and pyrrole is found in PAP4, it still shows the second highest results. We can also say that as the amount of Agar is increased, it resists the flow of ions and so the specific capacitance shows a decreasing trend. The amount of Agar in PAP3 is 5 mL and PANI/PPy electrodes were grown using 2.5 mL of aniline and pyrrole and it exhibited the smallest CV area and so the lowest specific capacitance. Here, we can deduce that although upon increasing the amount of Agar, the flow of ions is resisted, but here comes the influence of electrode materials i.e., if PANI/PPy is 2.5 mL it does not assist much in the transportation of ions however if the amount of PANI/PPy is increased to 5 mL, the results become comparatively better, which implies that the amount of electrode material here is 5 mL for PAP1 which contributes in the capacitive behavior. Hence, if 5 mL of Agar is used, it produces better results with 5 mL of PANI/PPy electrodes, i.e., PAP1. 

For the effect of electrode materials (PANI/PPy), the comparison of results showed that with 3 mL of Agar both 2.5 mL and 5 mL of electrode materials were used, but the results were better with 5 mL of PANI/PPy. On the other hand, when 5 mL of Agar was paired with both 3 mL and 5 mL of PANI/PPy, the results here were also promising with 5 mL of PANI/PPy as an increase in the capacitive performance was observed since it enhanced the conductivity of the capacitors. However, the overall promising performance was exhibited by PAP2 where 5 mL of the polymerized electrode materials made a good combination with 3 mL of Agar and 5 mL of PVA. These results were confirmed by performing GCD and other analysis and characterization that were conducted. Therefore, the effect of electrolyte and electrode can be distinguished.

#### 3.3.2. Galvanic Charge-Discharge

**Figure 11:**
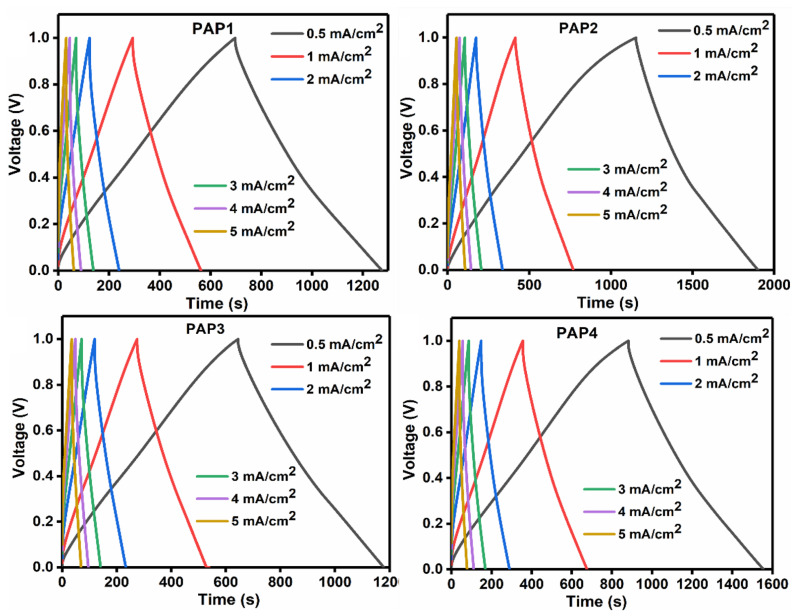
GCD curves of all samples at different current densities.

Galvanostatic charge-discharge (GCD) is a standard technique to study the electrochemical behavior and the cycle life of energy storage devices based on hydrogel electrolytes, by varying the currents. The device performance, therefore, was further studied by carrying out the galvanic charge-discharge (GCD) studies at different current densities ranging from 0.5 mA/cm^2^ to 5 mA/cm^2^. The results of the GCD studies are shown in Figure 11.

We can see that as current density is increasing, the discharge time of the device is decreasing. This happens because when the current density is higher, the ions present in the electrode and the electrolyte find a very limited time to interact with each other. Therefore, at low current densities, the ions have enough time to penetrate throughout the configuration due to which they get longer discharge time for the device [21]. Figure 11 shows the longest discharge time at a current density of 0.5 mA/cm^2^ which indicates that it has the maximum areal capacitance and is also capable of holding the charges for a longer time. The areal capacitance it exhibited was recorded to be 750.13 mF/cm^2^, which is finely agreeing with the CV results at 5 mV/s.

**Figure 12:**
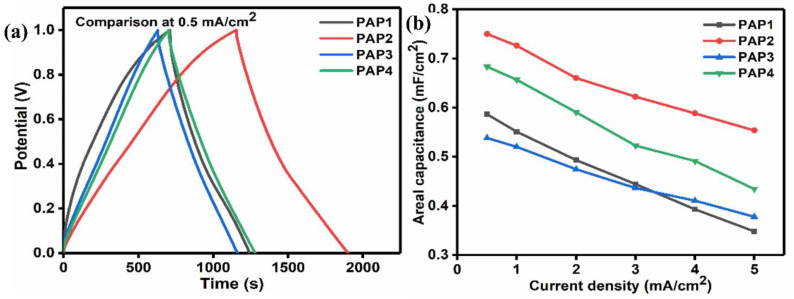
(**a**) Comparison of GCD curves at 0.5 mA/cm^2^, (**b**) areal capacitance vs. current density.

Figure 12a presents the comparison of GCD curves at a current density of 0.5 mA/cm^2^ where the symmetric triangular curves reveal the ideal reversible capacitance characteristics along with a small internal resistance known as the equivalent series resistance (ESR). A significant sharp but very small IR_drop_ is also observed. Another factor which is observed is a rapid increase in the start of the charging process. This happens because of two reasons; one is the direct effect of ESR, and the other reason is the characteristic redistribution of charges within the pores. The linear curves of charge and discharge also exhibit a good contact between conducting polymer electrodes and hydrogel electrolytes. Using these charge discharge curves; areal capacitance of the device has been calculated. These findings clearly reveal that the charge and discharge time and the specific capacitance depend on the current density and the composition of all-in-one configuration energy storage devices. At a current density of 0.5 mA/cm^2^, PAP2 shows an areal capacitance, energy density, and power density of 750.13 mF/cm^2^, 103.02 μWh/cm^2^ and 497.22 μW/cm^2^ respectively, having the maximum discharge time as compared to PAP4 which has the same amount of Agar i.e., 3 mL but contains a different number of polymerized electrodes i.e., 2.5 mL each. Discharge time comparison is shown in Figure 12a. This shows the effect of in situ grown conducting polymer electrodes, which contributed to the increase in the ion transportation, which in turn favored the specific capacitance and discharge time of the cell. By increasing the amount of Agar to 5 mL in PAP1, where PANI/PPy were 5 mL each, the specific capacitance and discharge time start decreasing. Then, in PAP3 where it was 5 mL of Agar, when the amount of conducting polymers PANI/PPy were decreased to 2.5 mL each, the discharge time and capacitance decreased even more, as discussed earlier. Figure 12b shows the plot of areal capacitance versus current density of all samples. This plot clearly shows the decrease in areal capacitance as the current density is increased. It indicates the proper activation of the polymerized conducting polymer electrodes along with the effect of varying the amount of Agar and conducing polymers PANI and PPy. Moreover, the contribution of electrode surface wettability with 5 mL of PANI and PPy each can be distinguished. 

**Figure 13:**
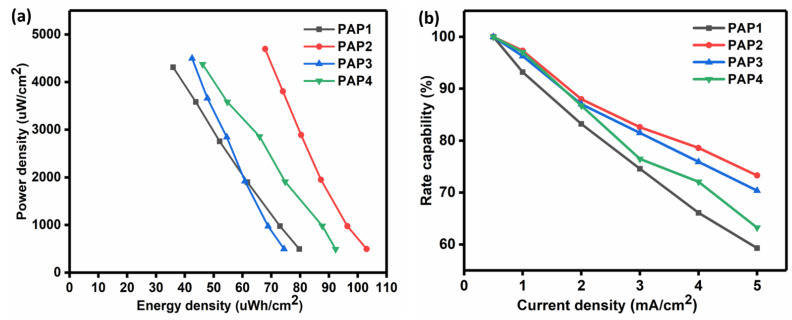
(**a**) Ragone plots of all supercapacitors and (**b**) Plot of rate capability against the current density.

Figure 13a represents the Ragone plot of the calculated power density versus the calculated energy density of all the samples. PAP2 delivered higher power density and energy density as compared to the other samples. By comparing PAP2 and PAP4, as both of them contain 3 mL Agar, we can see a clear difference in the energy density and power density. This difference is attributed to the in situ grown conducting polymer electrodes. The fact is that the best sample which is PAP2 contains 5mL of PANI and PPy each which improves the charge conductivity, while PAP4 contains 2.5 mL PANI and PPy each. Table 2 presents the maximum areal capacitance values, energy density, power density, and rate capability of all the samples that were obtained.

The attained energy density of PAP2 is higher compared to the reported pure PANI based conducting hydrogel based PANI-CPH (42 $\mu \mathrm{Wh}/{\mathrm{cm}}^{2})$ and PANI-PHP (42 $\mu \mathrm{Wh}/{\mathrm{cm}}^{2})$ all-in-one supercapacitors [20,22] (K. Wang et al., 2015), (Zhang et al., 2019) and pure PPy based PPy/CPH (23 $\mu \mathrm{Wh}/{\mathrm{cm}}^{2})$ and PPy/B-PVA/KCl (20 $\mu \mathrm{Wh}/{\mathrm{cm}}^{2})$. This higher energy density of all-in-one supercapacitor in this work is owing to the combined effect of PANI and PPy in the PAP2 [23,24].

The flexible all-in-one configuration supercapacitor which was fabricated using PAP2 showed a good rate capability of 73.3% as shown in Figure 13b. The porosity in the structure of the hydrogel electrolyte favored the transportation of ions, which decreased the resistance of the electrolytes’ ions found within the pores. This increased the rate capability. We can see that maximum rate capability is exhibited by PAP2. The rate capability decreased with a change in the composition and with an increase in the current density. Moreover, the rate capability of the PAP2 is greater than polyaniline or polypyrrole based supercapacitors where it is in the range of 50–70% [23,25]. This superior rate capability is owing to the effective ionic and charge transportation within the three-dimensional porous network of hydrogel in this work.

#### 3.3.3. Electrochemical Impedance Spectroscopy and Life Cycle Studies

**Figure 14:**
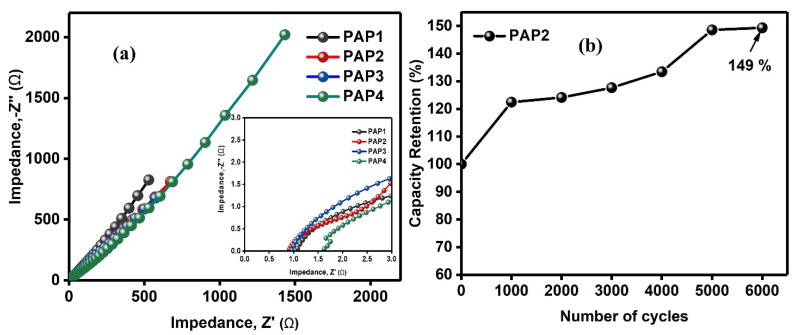
(**a**) Nyquist plots of all supercapacitors and (**b**) Cyclic stability of PAP2 all-in-one flexible supercapacitor (inset in figure demonstrate the Nyquist plot).

As the specific areal capacitance depends on the charge storage capacity of the electrode materials and charge transport. Therefore, electrochemical impedance spectroscopy (EIS) was performed. EIS is a technique to characterize the electroactive materials for their applications in energy storage devices. EIS explains the phenomena of ions movement towards or away from electrodes in a specific frequency range and output response is recorded in form of Nyquist plot. Herein, EIS results were plotted as Nyquist plots (Figure 14a) where the plots demonstrate negligible arcs at high frequency region and vertical lines at low frequency region. The negligible arcs show the minimal resistance faced by the ions and good capacitive behavior. The ohmic resistance (R_s_) and charge transfer resistance (R_ct_) were determined. The R_ct_ of 1.83, 0.45, 2.81, and 1.05 Ω for PAP1, PAP2, PAP3, and PAP4, respectively. The R_ct_ value depended on the composition of cells as it increased with the increase in contents of Agar and decrease in the contents of PANI/PPy. The increase in the Agar content resulted in the resistance in the movement of ions and reduction in the PANI/PPy contents could impede the transfer of electrons at the electrode-electrolyte interface. These R_ct_ results are in good agreement with the results of CV and GCD analyses.

The fabricated supercapacitor cells were flexible because not only hydrogels were used as electrolytes but also the electrodes were in situ polymerized on the hydrogel electrolytes’ surfaces. These electrodes were binder free so there was no extra dead weight on the cell. The overall configuration of the supercapacitor cells did not have a laminated configuration which makes them flexible. Owing to this fact, these supercapacitor cells can be employed in a wide range of applications for instance these can be used as smart technology in the electronics industry and flexible gadgets like watches. Figure 14b demonstrates the life cycle study of the highly performed PAP2 all-in-one flexible supercapacitor. The results reveal the increase in capacitance with the number of cycles owing to the activation of conducting polymers which resulted in the perforation of ions within the pores of conducting polymers from the electrolyte. This trend is frequently reported in literature for conducting polymer-based electrode materials [26,27]. PAP2 was investigated for 6000 cycles where it retained 149% capacitance. These results show the high stability of the supercapacitor which make it suitable candidate for wearable electronic applications [28]. The comparison of our work with the reported work is presented in Table 3.

## 4. Conclusions

Summarizing the work, we have successfully fabricated flexible all-in-one configuration supercapacitors, which showed excellent flexibility, areal capacitance, and mechanical robustness. We prepared four different samples to compare the results. The electrolytes of the supercapacitors were prepared by incorporating 5 wt.% PVA with different amounts of 5 wt.% Agar. In-situ polymerization mechanism was used to grow PANI/PPy electrodes by using aniline and pyrrole on the PVA/Agar hydrogel electrolytes. The synthesized all-in-one hydrogels were successfully characterized using FTIR, XRD, and FESEM. The mechanical strength was investigated through tensile testing. Then, all-in-one configured supercapacitor cells were fabricated by sandwiching the PANI/PPy-(PVA/Agar)-PANI/PPy hydrogels between two graphite sheets and the electrochemical performance studies were then carried out. The cyclic voltammetry (CV) and galvanic charge-discharge (GCD) of the assembled supercapacitors were carried out, which demonstrated that the prepared devices showed good specific capacitance and rate performance. One important factor which was studied was the effect of different amounts of Agar in the preparation of the electrolytes for different samples. Also, the effect of different quantities of aniline and pyrrole in the in situ polymerization of electrodes in different samples was observed. Using binder free electrodes by this in situ growth of polyaniline and polypyrrole together as electrodes on a hydrogel is a novelty in this work and these two made a good combination together and provided the supercapacitor with good electrochemical performance; therefore, the objective of the study has been achieved. The supercapacitor fabricated using PAP2, PANI/PPy-(PVA/Agar)-PANI/PPy conducting polymer hydrogel having 3 mL of Agar and 5 mL of PANI and PPy each showed the highest areal specific capacitance of 750.13 $\mathrm{mF}/{\mathrm{cm}}^{2}$ with an excellent energy density and power density of 103.02 $\mu \mathrm{Wh}/{\mathrm{cm}}^{2}$ and 497.22 $\mu W/{\mathrm{cm}}^{2}$, respectively. Moreover, R_ct_ results in EIS analysis demonstrate the minimum resistance was faced during the charge transfer in PAP2 and these results are in line with the CV and GCD investigations. The life cycle study presents the capacitance retention of 149% after 6000 cycles. We expect that the results of this work can be an inspiration for using new materials, which are unprecedented in the preparation of hydrogel electrolytes for supercapacitors, which can help in improving the performance of supercapacitors. We hope that this study will create new hubs of information and knowledge that would eventually generate new ideas.

## Figures and Tables

**Table 1:** Composition of all samples.

Formulation	PVA 5 wt.%	Agar 5 wt.%	1 M H_2_SO_4_	GA 4 *v*/*v*%	Aniline 1 M	Pyrrole 1 M	APS 1 wt.%
**PAP1**	5 mL	5 mL	2 mL	1 mL	5 mL	5 mL	2 mL
**PAP2**	5 mL	3 mL	2 mL	1 mL	5 mL	5 mL	2 mL
**PAP3**	5 mL	5 mL	2 mL	1 mL	2.5 mL	2.5 mL	2 mL
**PAP4**	5 mL	3 mL	2 mL	1 mL	2.5 mL	2.5 mL	2 mL

**Table 2:** The maximum performance properties obtained by the developed samples.

Results	PAP1	PAP2	PAP3	PAP4
Areal capacitance ($\mathrm{mF}/{\mathrm{cm}}^{2})$	586.75	750.13	538.39	683.43
Energy density $(\mu \mathrm{Wh}/{\mathrm{cm}}^{2}$)	79.62	103.02	74.38	92.31
Power density ($\mu W/{\mathrm{cm}}^{2}$ )	494.00	497.22	495.92	493.10
Rate capability $\left(\%\right)$	59.32	73.30	70.37	63.23

**Table 3:** Shows some of the reported work on polyaniline based hydrogels [29].

Material	Reported Specific Capacitance
PVA–PANI prepared by physical mixing	11.3 mF/cm^2^
PVA–PANI prepared by freeze–thawing method	420 mF/cm^2^
N-doped nanocarbon material derived from PANI	25.7 mF/cm^2^
PVA–PANI hydrogel	488 mF/cm^2^
Our work	750.13 mF/cm^2^

## Data Availability

Not applicable.
